# Coexisting nutcracker phenomenon and refractory hypertension in a patient with IgA nephropathy: A case report and literature review

**DOI:** 10.1002/ccr3.9542

**Published:** 2024-11-13

**Authors:** Fengmei Wang, Xinru Li, Ran Liu, Yao Wang, Lili Liu, Xiaoliang Zhang, Bicheng Liu

**Affiliations:** ^1^ Institute of Nephrology, Zhong Da Hospital Southeast University School of Medicine Nanjing Jiangsu China; ^2^ School of Medicine Southeast University Nanjing Jiangsu China; ^3^ Institute of Endocrinology, Zhong Da Hospital Southeast University School of Medicine Nanjing Jiangsu China

**Keywords:** hypertension, IgA nephropathy, left renal vein, nutcracker syndrome

## Abstract

The entrapment of the left renal vein (LRV) may contribute to changes in hemodynamics within kidney and could also be associated with IgA nephropathy (IgAN). Although the relationship between the nutcracker phenomenon and IgAN has not yet been elucidated, it is speculated that this patient's refractory hypertension is a combined effect of nutcracker syndrome (NCS) and IgAN.

## 1 INTRODUCTION

Left renal vein (LRV) entrapment is mainly divided into two types as follows: LRV entrapment between the superior mesenteric artery (SMA) and the abdominal aorta (AO), and compression between the AO and the spine.^1^ When clinical manifestations of LRV entrapment occur, it is called nutcracker syndrome (NCS). The most common renal presentations of NCS include hematuria and proteinuria.^2^ Other uncommon symptoms may also be accompanied, including abdominal pain, varicocele of the ovarian or spermatic veins, dyspareunia, dysmenorrhea, fatigue, and orthostatic intolerance and so on.^3^ The Genetic and Rare Disease Information Center at the National Institutes of Health (NIH‐GARD) has registered it as a rare disease, and the exact prevalence of NCS is unknown.^4^

Herein, we report a young woman in preparation for pregnancy who was incidentally diagnosed with NCS. Her urine routine found only microscopic hematuria and no proteinuria, which was common in NCS. Unusually, her main symptom was unmanageable hypertension, which fluctuated between 171 and 152/114‐96 mmHg. Detailed examination of common causes associated with hypertension was performed. However, no evidence was found. The patient further underwent abdominal contrast‐enhanced CT and vascular ultrasonography, revealing entrapment of the left renal vein. Combined with her clinical manifestations, this patient was finally diagnosed as NCS. Previous case reports of NCS associated with hypertension are rare.^5^ Hypertension caused by NCS is attributed to renovascular factors.^6^ In addition, our previous research found that the prevalence of IgAN in patients with NCS was significantly higher than in those with other glomerular diseases.^7^ Therefore, we performed a renal biopsy on this patient. The pathological results were the same as our speculation: the mild proliferation of mesangial cells and stroma with abundant IgA deposition.

## 2 CASE HISTORY

A slender 25‐year‐old woman was admitted to our hospital due to a 5‐day hypertension, with the highest blood pressure reaching 166/114 mmHg. Three separate blood pressure measurements on different days were all greater than 140/90 mmHg. Consequently, she came to our renal department for further consultation. She had no previous history of hypertension and no family history.

On admission, her body mass index (BMI) was 18.75 kg/m^2^. A physical examination revealed a blood pressure (BP) of 153/109 mmHg. No other obvious abnormality was found. The serum total protein level and albumin are 71.1, 47.3 g/L, respectively. In addition, the functions of liver and kidney are also normal. Urinalysis revealed microscopic hematuria (2.10 × 10^4^/mL) and minimal proteinuria. Laboratory examination showed a slight increase in 24‐h urine protein of 0.272 g (normal range: 0.028–0.141 g/24 h), increases in plasma renin activity (2.66 ng/mL/h, normal range: supine, 0.15–2.33 ng/mL/h) and angiotensin II (71.4 pg/mL, normal range: supine, 25–60 pg/mL). Plasma metanephrine, normetanephrine, and dopamine levels were reported as within normal limits, but in urine, these indicators were a little higher.

## 3 METHODS

In order to exclude endocrine factors of secondary hypertension, abdominal contrast‐enhanced CT and renal artery computed tomography angiography (CTA) were performed. The results showed that there was no obvious abnormality in bilateral adrenal glands, an adrenal tumor was not detected, and both renal arteries were intact.

**FIGURE 1:**
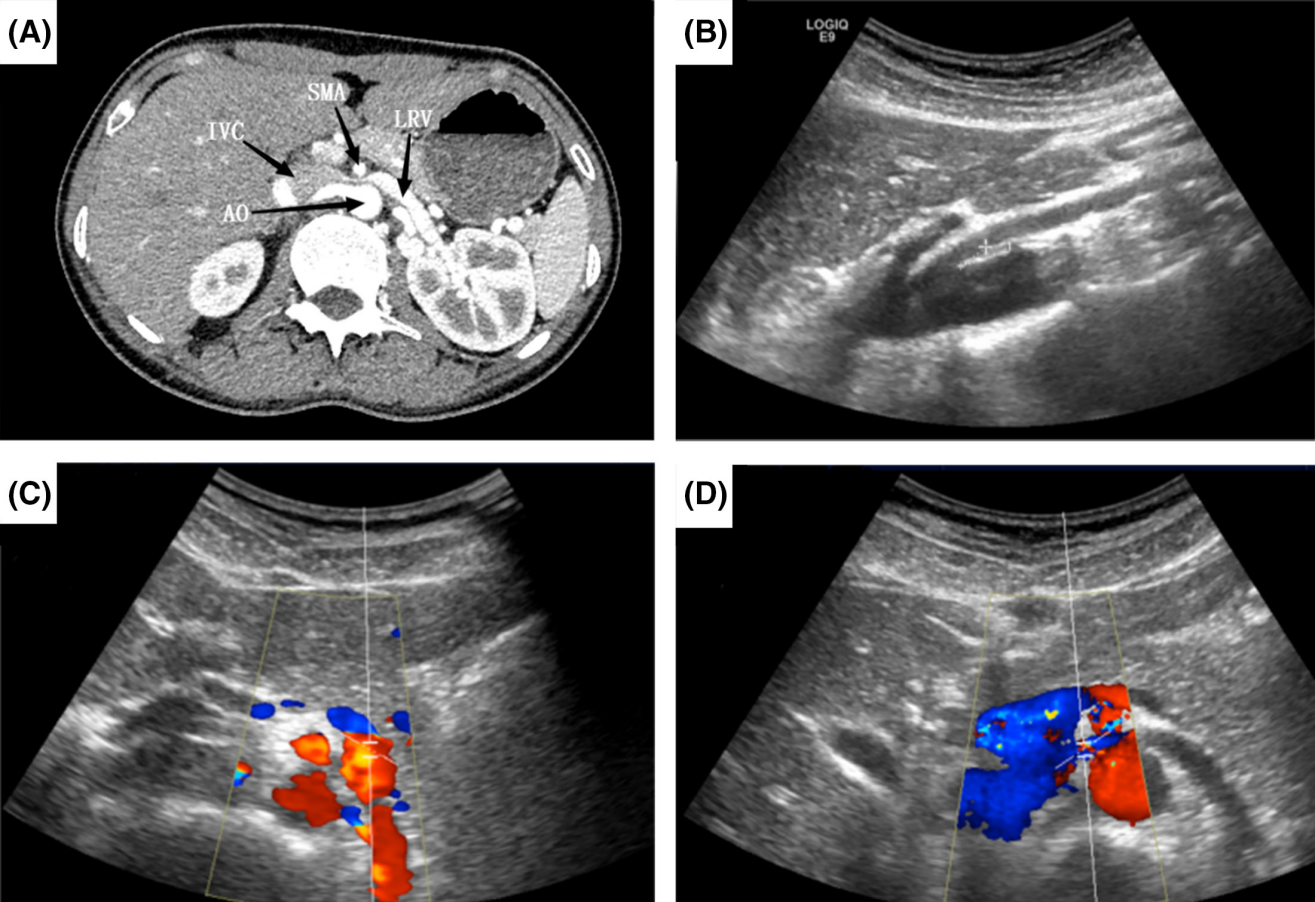
Imaging findings. (A) Abdominal contrast‐enhanced CT showed compression of the LRV between the SMA and the abdominal AO. (B–D) Color Doppler ultrasonography showed compression of the LRV between the SMA and the abdominal AO. (B) Two‐dimensional ultrasonography image. The angle between AO and SMA is 19.1°. (C) Color doppler ultrasonography flow image. TAMAX of the LRV trunk is 40.4 cm/s. (D) Color doppler ultrasonography flow image. TAMAX of LRV across the abdominal AO is 117.2 cm/s. CT, computed tomography; LRV, left renal vein; AO, abdominal aorta; SMA, superior mesenteric artery; IVC, inferior vena cava; LRV, left renal vein; AO, abdominal aorta; SMA, superior mesenteric artery; TAMAX, time‐averaged maximum velocity.

The unexpected discovery was that the LRV was compressed between the abdominal aorta and superior mesenteric artery (Figure 1A). Color Doppler ultrasonography also confirmed LRV entrapment (Figure 1B–D). As we all know, the anatomic abnormalities could lead to hemodynamic changes in the setting of NCS, which could deteriorate the fluctuation of hypertension.

What is more, the antihypertensive effect of taking amlodipine (5 mg once daily; Pfizer, America) orally was poor, and her blood pressure fluctuated within 171–152/114–96 mmHg.

**FIGURE 2:**
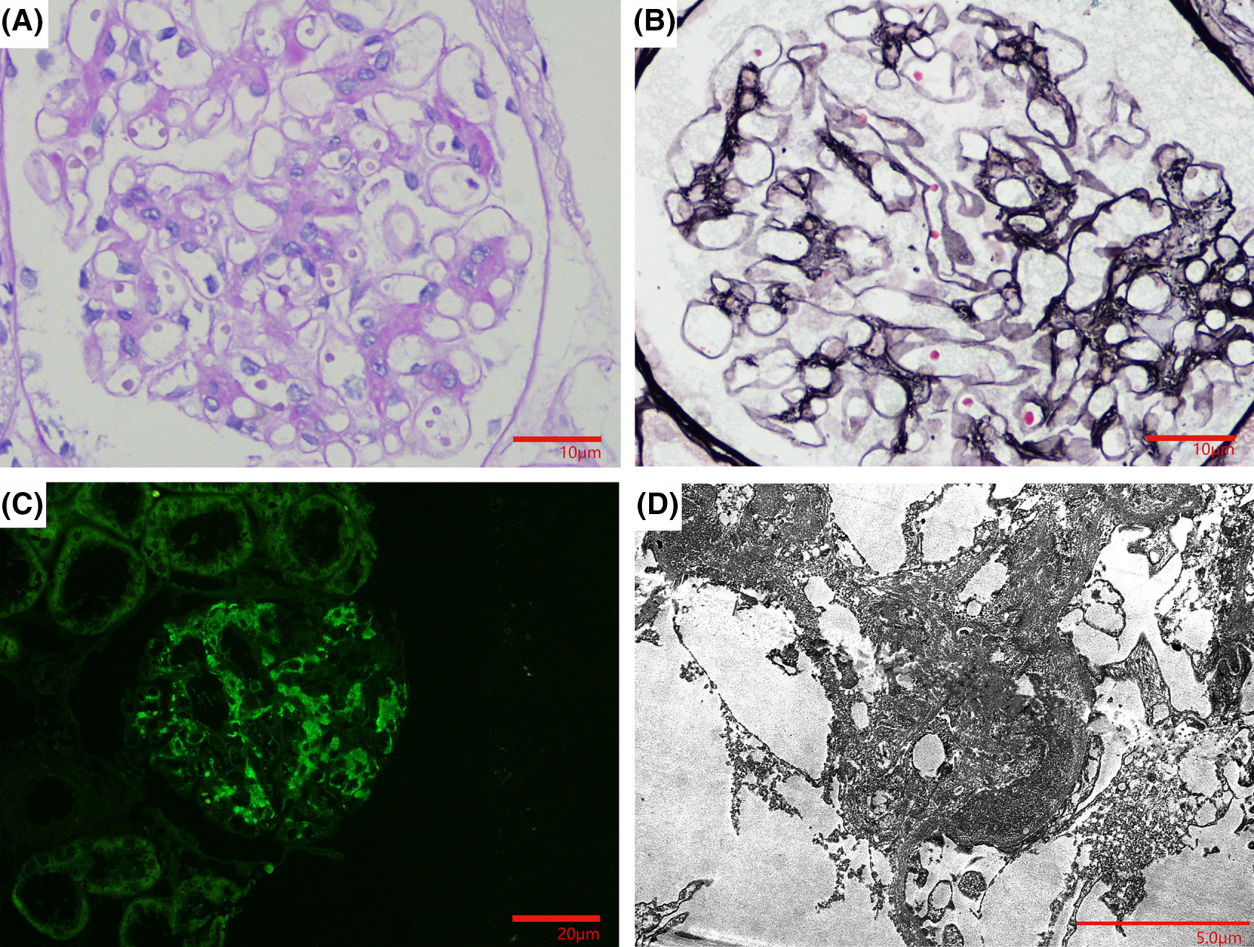
Renal biopsy findings. (A) Light microscopy demonstrated mild diffuse proliferation of glomerular mesangial cells and matrix, moderate to severe proliferation of focal segments. (B) Periodic Acid‐Schiff (PAS) staining demonstrated deposition of eosinophil in the mesangial area, mild renal tubulointerstitial lesions, focal atrophy of renal tubules. (C) Immunofluorescence microscopy showed the deposition of IgA in the mesangial area. (D) Periodic Acid‐Silver‐Methenamine (PASM) staining demonstrated electron micrograph demonstrated the deposition of electron‐dense material with different sizes in the mesangial area, accompanied by partial foot process fusion.

Based on the LRV entrapment and laboratory examination with hematuria and proteinuria, the diagnosis of NCS can be made. Then, a kidney biopsy was performed. Twenty glomeruli were sampled for light microscopy. Light microscopy showed mild diffuse mesangial hypercellularity and expanded mesangial matrix, as well as mild tubulointerstitial lesions (Figure 2A,B). Immunofluorescence revealed positive IgA (+++), IgM (+−), C3 (+−++), and negative others (Figure 2C). Electron microscopy revealed mounds of electron‐dense material of different sizes in the mesangial area, accompanied by partial foot process fusion (Figure 2D). Ultimately, a diagnosis of IgA nephropathy (IgAN) (M1E0S0T0C0) was established.

Then, the patient took Sacubitril Valsartan Sodium (100 mg once daily; Novartis, Switzerland) and amlodipine (5 mg once daily; Pfizer, America) to control blood pressure, and her blood pressure fluctuated between 132 and 106/73–80 mmHg. Due to the teratogenic side effect of Angiotensin‐Converting Enzyme Inhibitor (ACEI) on the fetus, the Novartis medication was withdrawn, and Labetalol (100 mg twice daily) instead. Unfortunately, her blood pressure rebounded again and fluctuated between 140 and 145/100–102 mmHg. After that, she switched back to oral ACEI, and her blood pressure was effectively controlled again. Hereto, we speculated that both NCS and IgA nephropathy were attributed to the refractory hypertension.

## 4 CONCLUSION

Although the association between the nutcracker phenomenon and IgA nephropathy has not been elucidated yet, the combination of anatomic abnormality and IgA nephropathy may promote refractory hypertension.

## 5 DISCUSSION

The anatomical phenomenon of left renal vein entrapment was first proposed in 1950,^8^ also known as nutcracker phenomenon (NCP). When NCP causes clinical manifestations, it is called nutcracker syndrome.^2^ There is no exact data on the prevalence of NCP. In recent years, with the improvement of diagnostic level, the prevalence rate is on the rise.^4^ The age distribution of NCP shows an obvious bimodal phenomenon, with most of which occur in adolescence and adulthood (in the 20th and 30th years), and is prone to relapse in the 30th and 40th years.^9^ NCP is also associated with body mass index, region (Asia), and gender (female).^10^ The prevalence is higher in people with low body mass index. There are no unified diagnostic criteria for NCP. Currently, the main standards for ultrasound diagnosis of NCP are the same as in our previous research: (1) the angle between the SMA and the abdominal aorta is less than 30°; (2) the flow velocity of stenosis of the LRV in the supine position accelerates remarkably; (3) the inner diameter ratio between the renal hilum and stenosis of the LRV in the supine position is >3; (4) the LRV entrapment with collateral circulation in the left lumbar ascending vein.^7^ Clinical manifestations of NCS can be divided into renal presentations and non‐renal presentations.^11^ The common renal presentations are hematuria and/or albuminuria, which are considered to be related to the increased pressure of the LRV and hemodynamic changes.^12^

Renal manifestations of this patient were microscopic hematuria and proteinuria. Interestingly, renal biopsy revealed IgAN. Our previous research found that the association between LRV entrapment and IgA nephropathy was not accidental. We retrospectively analyzed 797 patients with glomerular diseases diagnosed by renal biopsy in our center and other centers from January 2019 to August 2021. Data analysis revealed that the incidence of LRV entrapment was significantly higher in IgAN and IgA vasculitis (32/187, 17.1%) than in other pathologic types (15/610, 2.4%). This was suggested that LRV entrapment coexisted with several kinds of renal diseases, with a significantly higher prevalence in patients with idiopathic IgAN and Henoch–Schönlein purpura nephritis (HSPN).^7^ At present, there are few large sample reports on LRV entrapment combined with glomerular disease in the world, but a number of case reports of LRV entrapment combined with IgAN have been published.^13^, ^14^, ^15^ Naohiko et al. found that LRV entrapment was 6.8% of 146 patients with IgAN.^16^ And in our previous study, glomerular incidental IgA deposition was observed to be significantly more common in patients with LRV entrapment compared with to those without it. Furthermore, in glomerular diseases with incidental IgA deposits, a significantly larger proportion of patients with LRV entrapment were positive for glomerular Gd‐IgA1 in contrast to patients without LRV entrapment.

Thus, it is reasonable to speculate that there might be an unknown causal relationship between LRV entrapment and IgAN. But the mechanism is unclear and there is a lack of large sample studies, so further research is needed in the future.^15^, ^16^

So far, Gd‐IgA1 deposition has been identified as one of the core mechanisms in the pathogenesis of IgAN. The exact site of Gd‐IgA1 production remains unclear. However, the close relationship between the intestinal mucosal immune response and Gd‐IgA1 production has been supported by more and more studies.^17^ Through double immunofluorescent staining of Gd‐IgA1 and IgA, we noted that Gd‐IgA1 deposition was present in the renal tissues of patients with LRV entrapment accompanied by nephropathy. Moreover, in our follow‐up cohort, most patients were often accompanied by obvious gastrointestinal symptoms. On abdominal ultrasound, the SMA and AO compressed the duodenum at the same time. Combined with the previous results, we speculated that hemodynamic factors caused by LRV entrapment, may aggravate the development of renal injury, and relevant studies are currently underway.

Another interesting finding is that this patient had difficult‐to‐control hypertension. Hypertension is an uncommon non‐renal presentation of NCS, and the clinical search for secondary hypertension often ignores NCS. Renal vascular factor is one of the important factors of secondary hypertension. The 2018 European Society of Cardiology (ESC) Guidelines for Hypertension Management listed the common disorders of secondary hypertension, with renal vascular factors ranking third. The data showed that hypertension caused by renovascular factors accounts for 1%–10% of total hypertension.^18^ The main causes of renovascular hypertension are atherosclerotic renal artery stenosis and fibromuscular dysplasias.^19^ Hypertension caused by NCS is rare. Case reports of hypertension caused by NCS are rare. In 2014, Se‐Jun Park et al. reported a case of hypertension caused by NCS with a BMI of 19.5 and ovarian varices.^20^ Similar to our case, both patients had increased renin levels and failed to use calcium channel blocker (CCB) to decrease blood pressure. However, after taking ACEI, both patients' hypertension was effectively controlled. In addition, Yoko Hosotani et al.^21^ and Wang RF et al.,^21^ also reported renin‐elevated NCS cases with hypertension, whose blood pressure returned to normal after surgical relief or the use of ACEI.

Therefore, some researchers believe that hypertension in patients with NCS may be due to increased pressure in the LRV caused by LRV entrapment, which stimulates the increased release of renin.^21^, ^22^ Increased renin secretion activates the renin‐angiotensin‐aldosterone system (RAAS) to cause water‐sodium retention. Subsequently, the body's blood volume increases, causing blood pressure to rise.^23^ This mechanism could explain the normalization of blood pressure after ACEI treatment in NCS‐related hypertension. This patient's blood pressure decreased significantly after using ACEI, and rebounded again with hematuria and proteinuria after drug withdrawal. Compared with other patients of significant renin elevation, her elevated levels of serum renin were limited but accompanied by ovarian varices suggestive of severe venous pressure. This may suggest that local renin level in local left kidney may be higher than serum.

However, this mechanism does not explain all hypertension in NCS. For instance, renin level was normal in patient reported by Narkhede et al. in 2017^24^; The patient Hadei et al. reported in 2020 even had a lower renin level.^25^ Mechanisms of hypertension are very complex in the setting of NCS. Some researchers believe that renal venous hypertension can cause elevated renal interstitial pressure, renal arterial vasculature myogenic response, neural vasoconstrictive reflexes, baroreceptor activation, sympathetic nervous system (SNS) activation, glomerular hyperemia and sclerosis, and ischemia and hypoxia‐related inflammatory injury.^26^ These factors could contribute to a decrease in glomerular filtration rate (GFR), resulting in water‐sodium retention and elevated blood pressure.

In addition, the patient was also diagnosed with IgAN, which is the most common glomerular disease.^27^ IgAN induced hypertension belongs to renal parenchymal hypertension, causing renal parenchymal ischemia and hypoperfusion, activating RAAS system, and leading to water and sodium retention. Studies have shown that IgAN is one of the common causes of hypertension. Meanwhile, hypertension is also a major risk factor for progression in IgAN patients, which can affect the 5–10 years survival rate.^28^ Based on clinical manifestations, examination results, and changes after treatment of this patient, it is reasonable to speculate that hypertension and renal injury may not only be related to hemodynamics caused by LRV entrapment, but also will be aggravated by chronic glomerular disease caused by non‐hemodynamic factors. Therefore, it is necessary to pay attention to the relationship between LRV entrapment and chronic glomerular diseases, which is beneficial to early diagnosis of kidney disease and the delay of kidney injury.

## AUTHOR CONTRIBUTIONS

**Fengmei Wang:** Data curation; writing – original draft; writing – review and editing. **Xinru Li:** Writing – original draft. **Ran Liu:** Writing – original draft. **Yao Wang:** Data curation. **Lili Liu:** Data curation. **Xiaoliang Zhang:** Conceptualization; writing – review and editing. **Bicheng Liu:** Conceptualization; writing – review and editing.

## FUNDING INFORMATION

This work was supported by grants from the National Natural Science Foundation of China (82060132), Zhongda Hospital Affiliated to Southeast University, Jiangsu Province High‐Level Hospital Pairing Assistance Construction Funds(zdyyxy02) and Zhongda Hospital Affiliated to Southeast University, Jiangsu Province High‐Level Hospital Pairing Assistance Construction Funds (zdlyg02).

## CONFLICT OF INTEREST STATEMENT

The authors declare that the research was conducted in the absence of any commercial or financial relationships that could be construed as a potential conflict of interest.

## ETHICS STATEMENT

This study was approved by the Medicine Ethics Committee of Southeast University affiliated with Zhong Da Hospital. The patients/participants provided their written informed consent to participate in this study. Written informed consent was obtained from the proband, his parents, the paternal aunt and the sister for the publication of any potentially identifiable images or data included in this article.

## CONSENT

Written informed consent was obtained from the patient to publish this report in accordance with the journal's patient consent policy.

## Data Availability

Further clinical data and images of this case are available from the corresponding author upon reasonable request.
